# Impact of *Helicobacter pylori* Infection on Metabolic and Physiological Parameters Among Young Adults Individuals

**DOI:** 10.3390/jcm15114046

**Published:** 2026-05-23

**Authors:** Ashwag Alsharidah, Jehan Mohamed Abdelsalam Mansour

**Affiliations:** 1 Department of Physiology, College of Medicine, Qassim University, Buraydah 52571, Saudi Arabia; 2 Department of Microbiology Central Laboratories, Ministry of Health and Population (MOHP), Cairo 11516, Egypt

**Keywords:** *Helicobacter pylori*, young adults, C-reactive protein, erythrocyte sedimentation rate, glycemic control, HbA1c, iron deficiency anemia, Insulin resistance, ferritin

## Abstract

**Background/Objectives:***Helicobacter pylori* infection is traditionally associated with gastrointestinal diseases; however, increasing evidence suggests that it may have systemic effects involving inflammatory, metabolic, and hematological pathways. Despite this, integrated evaluations of these domains remain limited, particularly in Middle Eastern populations. This study aimed to assess the impact of *H. pylori* infection on inflammatory, metabolic, and hematological parameters among adults. **Methods:** A case–control study was conducted including 100 participants (50 *H. pylori*-positive patients and 50 healthy controls) recruited from Qassim Health Cluster, Saudi Arabia. Demographic and clinical data were collected, and blood samples were analyzed for random blood sugar (RBS), glycated hemoglobin (HbA1c), C-reactive protein (CRP), erythrocyte sedimentation rate (ESR), hemoglobin, ferritin, and white blood cell count (WBC). Statistical analyses included group comparisons, Spearman correlation, logistic regression, and receiver operating characteristic (ROC) curve analysis. **Results:** The infected group showed significantly higher levels of RBS and HbA1c, indicating impaired glycemic control. Inflammatory markers (CRP and ESR) were also significantly elevated compared to controls (*p* < 0.001). Hemoglobin and ferritin levels were significantly lower in the infected group (*p* < 0.001), suggesting disturbed iron metabolism. Correlation analysis revealed positive associations between inflammatory markers and glycemic indices, and negative associations with hemoglobin and ferritin. Multivariable logistic regression identified CRP (adjusted OR = 1.33, 95% CI: 1.04–1.71) and ESR (adjusted OR = 1.09, 95% CI: 1.02–1.16) as independent predictors of *H. pylori* infection after adjustment for smoking status and fast-food consumption. The combined model demonstrated acceptable discriminatory performance with an AUC of 0.82 (95% CI: 0.74–0.90). **Conclusions:** *Helicobacter pylori* infection was associated with significant inflammatory, metabolic, and hematological alterations, supporting its potential role as a systemic condition beyond the gastrointestinal tract. These associations remained significant after adjustment for major lifestyle-related confounders, including smoking status and fast-food consumption. Although the combined inflammatory model demonstrated acceptable discriminatory performance, it should currently be considered mainly for research or preliminary screening purposes and not as a replacement for established diagnostic methods for active *H. pylori* infection. Further large-scale longitudinal and interventional studies are warranted to clarify causality and evaluate the impact of eradication therapy on systemic outcomes.

## 1. Introduction

*Helicobacter pylori* is a Gram-negative bacterium, which is a spiral-shaped organism, that resides in the gastric mucosa (stomach lining). It is estimated that over 50% of the global population is infected [1]. Although many of the individuals infected will show no symptoms, *H. pylori* is a causal factor for chronic gastritis, gastric ulcers, gastric cancer, adenocarcinoma and MALT (mucosa-associated lymphoid tissue lymphoma) [2].

In addition to the H. pylori-related gastrointestinal diseases, it is now recognized that *H. pylori* is of a systemic nature that impacts organs outside of the GI tract [3]. *H. pylori* will cause chronic infection and persistent subclinical inflammation, which are caused by the proinflammatory cytokines, (IL-6) and TNF-a. These will cause a metabolic disturbance and lead to narrowed blood vessels (endothelial dysfunction). These inflammatory processes will cause the insulin pathways, specifically, the insulin receptor and glucose transport systems to be interrupted, and ultimately lead to insulin resistance [4].

The systemic effects of *H. pylori* infections on the gut–brain axis have been continuously expanding. *H. pylori* infection alters gastric and intestinal microbiota that can disrupt the neuroendocrine system, appetite control, and energy homeostasis, causing the development of metabolic diseases like obesity and type 2 diabetes [5]. Additionally, infected patients have been shown to have disruption of the gastric hormones, ghrelin and leptin, which suggests *H. pylori* infection could be related to some of the components of metabolic syndrome [6].

There have been some recent epidemiological and mechanistic studies that show a notable correlation between *H. pylori* infection and the deterioration of glycemic control. Song et al. [7] conducted a meta-analysis and found there to be a correlation between *H. pylori* infection and the development of insulin resistance. Furthermore, growing evidence from systematic reviews and meta-analyses indicates that *H. pylori* infection is associated with an increased risk of metabolic syndrome and insulin resistance [8]. From these studies it can be concluded that *H. pylori* infection is a contributor to the development of metabolic syndrome and therefore may be a target for interventions to improve metabolic control.

*H. pylori* infection affects patients’ metabolism and happens to coincide with another complication: iron deficiency anemia. Several explanations have been put forward. One of which is hypochlorhydria-associated iron malabsorption. This is followed by the bacteria consuming iron and the consequent chronic inflammation of the gastric mucosa which leads to slow but extensive and unnoticeable blood loss [9]. In addition, Fujii et al. [10] argue that in a chronic infection context, regulation of iron by hepcidin leads to more iron being locked up and a relative stoppage of production of red blood cells. This phenomenon is very similar to deficiency anemia of chronic ailments (2024).

Elevated levels of the aforementioned markers and consequent immune activation have been linked to metabolic and blood related disorders as described in [11]. On that note, discomfort from the *H. pylori* related infection has been captured in inflammatory markers like the sedimentation rate of erythrocytes (ESR) as well as in C-reactive protein (CRP). Some literature like Li et al. [12] suggested using these inflammatory markers in tandem with each other to improve the quality of diagnosis as opposed to using each of the markers in isolation for diagnosis.

Additionally, *H. pylori* infection may be a precipitating factor in the development of certain ACD and atherosclerosis-related conditions, such as endothelial dysfunction, due to the potential pathways of oxidative stress, chronic systemic inflammation, and immune-mediated injury to the vasculature [13]. This reinforces the hypothesis that chronic *H. pylori* infection is a condition that is systemic in nature and that its effects go far beyond the gastric area.

The literature continues to grow, but the relationships of *H. pylori* infection with the metabolic, inflammatory, and hematological factors are still poorly defined and inconsistent in the various studied populations. Especially in the Middle Eastern populations, Saudi Arabia in particular, the data are fewer, and the studies that evaluate the above parameters in a combined, interconnected, and sufficiently comprehensive analytical framework are few. Most of the previous studies have focused on a single factor of the pathology, without consideration of the interconnected metabolic, inflammatory, and hematological factors.

Thus, the goal of the current study is to assess the impact of *H. pylori* infection on the aforementioned metabolic, inflammatory, and hematological parameters in the adult population of Saudi Arabia. There is a need to integrate multiple study domains to evaluate the systemic outcome of *H. pylori* infection and to provide insight into its potential role as a modifiable factor in the associated metabolic and inflammatory disorders.

## 2. Materials and Methods

### 2.1. Study Design and Participants

This study was designed as a case–control study conducted among patients attending [General Hospital, Qassim Health Cluster, KSA]. A total of 100 participants were included, comprising 50 patients with confirmed *Helicobacter pylori* infection and 50 apparently healthy controls, matched for age and sex (18–60 years).

Sample size was calculated based on previously reported differences in hemoglobin concentration between *H. pylori*-positive and control participants [14]. The expected mean hemoglobin values were 13.3 ± 1.56 g/dL and 14.3 ± 1.89 g/dL, respectively, giving an expected mean difference of 1.0 g/dL. The pooled standard deviation was approximately 1.73, corresponding to an estimated moderate effect size (Cohen’s d = 0.58). Using a two-tailed test, 95% confidence level, and 80% statistical power, the minimum required sample size was estimated as 46 participants per group. Therefore, 50 participants were included in each group to allow for possible incomplete data.

Selection criteria for this study were: Adults between 18 and 60 years of age and positive for *H. pylori* infection (for the case group). Exclusion criteria were: Recent (1 month) use of antibiotics or PPI, History of surgery of the gastrointestinal tract, and Chronic diseases that interfere with the blood system.

Participants were categorized into case and control groups according to *H. pylori* infection status. Cases included participants with positive serological findings for *H. pylori* IgG/IgM antibodies using a rapid immunochromatographic assay. Controls were recruited from apparently healthy individuals attending the same healthcare setting and without documented history of *H. pylori*-related gastrointestinal disease. Controls were selected to achieve approximate matching for age and sex with the infected group. Fifty participants were enrolled in each group.

### 2.2. Tools

#### 2.2.1. Clinical and Anthropometric Assessment

Demographic data, clinical symptoms, dietary habits, and medication history were collected using a structured questionnaire developed by the researchers. Anthropometric measurements, including body mass index (BMI) and waist circumference, were recorded using standardized procedures.

#### 2.2.2. Specimen Collection and Processing

Venous blood samples (8 mL) were collected from all participants under aseptic conditions. 3 mL was collected in EDTA tubes for complete blood count (CBC) and HbA1c analysis. 5 mL was collected in plain tubes, allowed to clot, and centrifuged at 3000 rpm for 10 min to obtain serum.

#### 2.2.3. Biochemical Analysis

*Helicobacter pylori* infection was assessed using a rapid serological immunochromatographic assay for detection of *H. pylori* IgG/IgM antibodies. The test identifies exposure to *H. pylori* infection; however, it does not differentiate between active and past infection. Random blood glucose and HbA1c were measured using microparticle enzyme immunoassay (MEIA) (Abbott Diagnostics) (Abbott Park, IL, USA). Serum ferritin levels were determined using a colorimetric method (Stanbio Laboratory kit) (Boerne, TX, USA). C-reactive protein (CRP) levels were measured using a quantitative immunoassay method.

#### 2.2.4. Hematological Analysis

Hematological parameters, including white blood cells (WBCs), red blood cells (RBCs), hemoglobin (Hb), and platelets (PLTs), were analyzed using an automated hematology analyzer (Cell-Dyn 1700, Abbott Diagnostics, Abbott Park, IL, USA).

### 2.3. Ethical Considerations

The Bio and Med Ethics Committee approved the study protocol (Approval No. [H-04-Q-001]). The study complies with the guidelines of the Declaration of Helsinki. The participants were informed of the study’s concerns, procedures, risks, and benefits, and provided their written informed consent. Participants were free to withdraw from the study at any moment without any implications. Anonymity and confidentiality were upheld. Participants’ identifiable information was substituted with number codes, and data was securely stored and only used for the study. All the procedures were non-invasive, barring the routine blood sampling, and the highest safety precautions were observed during blood collection.

### 2.4. Field Work

The study’s fieldwork lasted 6 months, at the Qassim Health Cluster, General Hospital. Patients were randomly chosen, including healthy participants as control. The participants were explained the objectives and methods of the study, and then asked for consent. The participants were later interviewed, and their clinical and anthropometric data were collected. A trained nurse collected venous blood samples in aseptic conditions. Samples were collected, and examined, within the same day. The samples were examined using the standard methods for maximum reliability. The completeness of the study was ensured by data collection and control. The data collection and control were monitored by the team regularly.

### 2.5. Statistical Analysis

Data were analyzed using IBM SPSS Statistics version 23.0 (IBM Corp., Armonk, NY, USA). Quantitative data were expressed as mean ± standard deviation (SD) or median (range), as appropriate. Qualitative data were presented as frequencies and percentages. Comparisons between groups were performed using the Chi-square test for categorical variables and appropriate parametric or non-parametric tests for continuous variables. Spearman’s correlation coefficient was used to assess relationships between variables. Binary logistic regression analysis was conducted to identify independent predictors of *H. pylori*-associated alterations. Receiver Operating Characteristic (ROC) curve analysis was used to evaluate diagnostic performance, and the area under the curve (AUC) was calculated. A *p*-value < 0.05 was considered statistically significant.

Receiver Operating Characteristic (ROC) curve to draw roc curve; the true positive rate (Sensitivity) is plotted on (y) axis and false positive rate (100-Specificity) on (x) axis.

Receiver Operating Characteristic (ROC). Interpretation Categories of Area Under the ROC Curve (AUC) Values, Table 1.

## 3. Result

Table 2 presents the demographic and clinical characteristics of the studied groups. There were no statistically significant differences between the diseased and control groups regarding age (41.2 ± 13.1 vs. 36.3 ± 12.6 years, *p* = 0.062) or body mass index (27.86 ± 5.61 vs. 26.0 ± 4.56 kg/m^2^, *p* = 0.074), indicating comparability between the groups. Similarly, no significant differences were observed in sex distribution (*p* = 0.230), employment status (*p* = 0.160), or marital status (*p* = 0.260).

In contrast, smoking status differed significantly between the two groups (*p* < 0.001), with a higher proportion of smokers in the diseased group (34.0%) compared to none in the control group. Additionally, fast food consumption showed a statistically significant difference (*p* < 0.001), as participants in the diseased group reported higher frequencies of fast-food intake, whereas the majority of the control group reported no fast-food consumption (86.0%).

Overall, the findings suggest that while the two groups were comparable in baseline demographic characteristics, lifestyle factors particularly smoking and fast-food consumption were significantly associated with the diseased group.

Table 3 demonstrates the comparison of hematological and inflammatory markers between the diseased and control groups. The diseased group exhibited significantly higher levels of random blood sugar (112.36 ± 29.6 vs. 88.26 ± 7.64 mg/dL, *p* < 0.001) and HbA1c (5.90 ± 1.14 vs. 5.4 ± 0.67%, *p* = 0.009), indicating poorer glycemic control compared to controls. Inflammatory markers were also markedly elevated in the diseased group. Median CRP levels were significantly higher (5.45 vs. 2.75 mg/L, *p* < 0.001), as well as ESR (22.5 vs. 11 mm, *p* < 0.001), reflecting a pronounced inflammatory state.

Regarding hematological parameters, hemoglobin levels were significantly lower in the diseased group (12.3 ± 1.9 vs. 13.7 ± 0.93 g/dL, *p* < 0.001), suggesting a higher prevalence of anemia. Similarly, ferritin levels were significantly reduced in the diseased group (11.9 ± 2.40 vs. 30.5 ± 3.00, *p* < 0.001), supporting the presence of iron deficiency or altered iron metabolism. In contrast, no statistically significant difference was observed in white blood cell count between the two groups (median: 7.3 vs. 7.6 × 10^9^/L, *p* = 0.528). Overall, these findings indicate that the diseased group is characterized by significant metabolic dysregulation, heightened inflammatory response, and impaired hematological status compared to the control group.

Table 4 illustrates the Spearman correlation coefficients between inflammatory, glycemic, and hematological parameters among the studied participants. A statistically significant positive correlation was observed between CRP and ESR (r = 0.261, *p* = 0.009), indicating concordance between inflammatory markers. CRP also showed significant positive correlations with RBS (r = 0.227, *p* = 0.023) and HbA1c (r = 0.285, *p* = 0.004), suggesting an association between systemic inflammation and impaired glycemic control. Similarly, ESR demonstrated significant positive correlations with RBS (r = 0.385, *p* < 0.001) and HbA1c (r = 0.212, *p* = 0.035), further supporting the link between inflammation and metabolic dysregulation. In contrast, hemoglobin levels were significantly negatively correlated with both CRP (r = −0.213, *p* = 0.033) and ESR (r = −0.254, *p* = 0.011), indicating that higher inflammatory activity is associated with lower hemoglobin levels. Ferritin showed a significant negative correlation with ESR (r = −0.403, *p* < 0.001), while its correlation with CRP did not reach statistical significance (r = −0.180, *p* = 0.073). Overall, these findings demonstrate a significant interplay between inflammatory markers and glycemic indices, alongside an inverse relationship between inflammation and hematological parameters.

Logistic regression analysis (Table 5) was performed to identify significant predictors of *H. pylori* infection. In the univariate analysis, CRP, ESR, smoking status, and fast-food consumption were significantly associated with increased odds of infection. In the multivariable logistic regression model, after adjustment for smoking status and fast-food consumption, both CRP and ESR remained independent predictors of *H. pylori* infection. Elevated CRP levels were associated with increased odds of infection, while higher ESR values also showed a significant positive association with *H. pylori* positivity. Smoking status and frequent fast-food consumption additionally demonstrated independent associations with infection risk.

**Figure 1:**
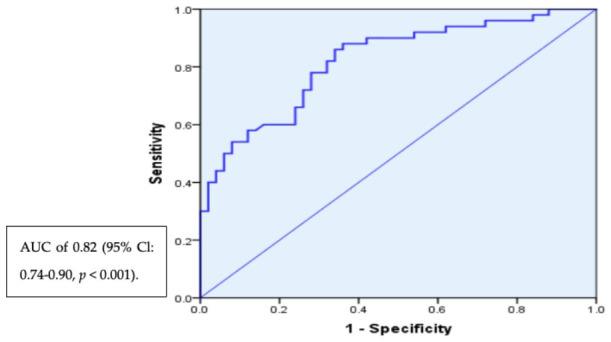
Receiver Operating Characteristic (ROC) Curve for Predicting *H. pylori* Infection.

Figure 1 Receiver operating characteristic (ROC) curve demonstrating the diagnostic performance of the combined CRP and ESR model for predicting *H. pylori* infection. The model demonstrated good discriminatory ability, with an area under the curve (AUC) of 0.82 (95% CI: 0.74–0.90; *p* < 0.001).

Table 6 summarizes the diagnostic performance of CRP, ESR, and their combined model in predicting *H. pylori* infection. CRP at a cutoff ≥ 6 mg/L demonstrated high specificity (90.0%) and positive predictive value characteristics, but low sensitivity (42.0%), indicating limited utility as a screening tool but usefulness in confirming disease. In contrast, ESR at a cutoff ≥ 12 mm/h showed high sensitivity (80.0%) but relatively low specificity (54.0%), suggesting its suitability as a screening marker rather than a confirmatory test.

The combined logistic regression model incorporating both CRP and ESR provided the best overall diagnostic performance, with an area under the curve (AUC) of 0.82 (95% CI: 0.74–0.90, *p* < 0.001), indicating good discriminative ability. At a probability threshold of 0.50, the model achieved a balanced performance with sensitivity of 60.0%, specificity of 76.0%, and overall accuracy of 68%. Overall, these findings suggest that while CRP and ESR individually have complementary diagnostic roles, their combination improves diagnostic accuracy and provides a more balanced approach for predicting *H. pylori* infection.

## 4. Discussion

The findings of the present study indicate that *Helicobacter pylori* infection is associated with multiple systemic alterations involving inflammatory, metabolic, and hematological parameters. Rather than being limited to localized gastric mucosal effects, *H. pylori* infection appears to contribute to chronic low-grade systemic inflammation with manifestations extending beyond the gastrointestinal tract. Evaluating these interrelated domains together may provide a more comprehensive understanding of the broader clinical impact of *H. pylori* infection, particularly in populations where integrated systemic assessment remains limited.

One of the most obvious results is a significant increase in inflammatory parameters, namely C-reactive protein (CRP) and erythrocyte sedimentation rate (ESR), for people with this infection. Indeed, as it has been known before, this pathogen can cause a prolonged immune reaction that can result in increased concentrations of pro-inflammatory cytokines including IL-6 and TNF-α. In their turn, these cytokines activate production of acute phase proteins by the liver including CRP [15,16]. At the same time, one should avoid misinterpretations of these results since the increase in CRP and ESR levels occurs due to any kind of inflammatory process. For that reason, their value lies more in supporting a broader clinical picture rather than serving as standalone indicators of infection.

Indeed, there is a connection between the examined condition and higher RBS and HbA1c. Such results indicate an association between *Helicobacter pylori* infection and impaired glycemic control. The results are consistent with the findings of recent research on inflammatory mediators’ ability to interfere with insulin signaling pathways, thus inhibiting glucose uptake and resulting in hyperglycemia [8,17]. Another area receiving increasing attention is the impact of inflammation on metabolic disorders due to disruption of normal gut microbiota and gastric hormones involved in regulating appetite and energy intake [18,19]. However, it should be noted that the majority of existing evidence, including the one provided above, is derived from cross-sectional analysis; therefore, longitudinal studies are necessary to establish the exact nature of causality.

In addition to the findings related to impaired glycemic control, it is also noteworthy that patients suffering from the infection experience reduced hemoglobin and ferritin. Such a result demonstrates the association between the examined condition and disrupted iron metabolism. Indeed, there is ample literature evidence indicating a link between *H. pylori* and iron deficiency anemia [20]. The mechanisms behind this link may include decreased acid production leading to poor iron absorption; competing for limited amounts of iron by the bacteria; gastric mucosa bleeding as a consequence of inflammation; increased levels of hepcidin leading to iron deficiency and suppressed erythropoiesis [10,11]. Nevertheless, given the size of the difference detected in ferritin levels, it is reasonable to assume that other factors might also play an important role.

The correlation analysis aims at integrating the data gathered throughout the experiment. The identified positive correlations between the indicators of inflammation and blood glucose levels, as well as negative correlations with hemoglobin and ferritin concentrations, indicate that the same physiological mechanisms operate during these processes. Inflammation can be viewed as an intermediate factor connecting metabolic disorders and bone marrow impairment. This trend corresponds with the recent studies of immunometabolism, where the connections between immune system activation and metabolism have been found [21].

The results of the regression analysis supported the initial hypothesis, as CRP and ESR remained independent predictors of *H. pylori* infection even after adjustment for smoking status and fast-food consumption. In addition, smoking and unhealthy dietary habits demonstrated significant associations with infection risk, highlighting the potential contribution of lifestyle-related factors to the observed inflammatory and metabolic alterations [8,19]. The combined multivariable model demonstrated better diagnostic performance than the use of individual inflammatory markers alone. These findings are consistent with previous literature suggesting that combining biomarkers and clinically relevant covariates improves diagnostic accuracy compared with isolated parameters [22,23]. Nevertheless, despite the improved predictive performance, these markers cannot replace established diagnostic methods for active *H. pylori* infection, such as urea breath testing or stool antigen assays [15].

The strengths of this study include the case–control design, relatively balanced study groups, and the use of multiple analytical approaches, including correlation and multivariable regression analyses. However, several limitations should be acknowledged. First, the use of serological testing may have resulted in misclassification bias because antibodies may persist after prior infection and cannot distinguish active from past infection [15]. Second, although smoking status and fast-food consumption were statistically adjusted for in the regression model, residual confounding related to dietary intake, micronutrient status, physical activity, and socioeconomic factors cannot be completely excluded. Previous studies have demonstrated that smoking and unhealthy dietary patterns may independently influence inflammatory markers, insulin resistance, and iron metabolism [18,24]. In addition, the relatively small sample size and single-center design may limit the generalizability of the findings.

From a clinical perspective, the findings suggest that the assessment of patients with *H. pylori* infection should extend beyond gastrointestinal manifestations alone. Evaluation of inflammatory status, glycemic control, and iron metabolism may provide additional insight into the broader systemic effects of the infection. Whether eradication therapy may improve these metabolic and hematological alterations remains an important question requiring further longitudinal and interventional studies [7,8].

To conclude, the paper adds on to the growing body of evidence regarding the association of *H. pylori* infection with the interconnecting dysfunctions related to inflammatory processes, metabolic activity, and blood properties. Even though the findings support existing biological knowledge, one cannot overlook the need for further research in order to establish the exact connections between the aforementioned factors and to prove if addressing the infection would yield improvements in patients’ well-being.

## 5. Conclusions

The present study demonstrates that *Helicobacter pylori* infection is associated with significant systemic alterations involving inflammatory, metabolic, and hematological parameters. Infected individuals exhibited elevated inflammatory markers, impaired glycemic control, and reduced hemoglobin and ferritin levels, supporting the concept that *H. pylori* infection extends beyond a localized gastrointestinal disorder. The observed associations between inflammatory markers, glycemic indices, and hematological parameters further suggest a complex interplay potentially mediated by chronic low-grade systemic inflammation.

Importantly, these associations remained significant even after adjustment for major lifestyle-related confounding factors, including smoking status and fast-food consumption, strengthening the evidence for an independent relationship between *H. pylori* infection and systemic abnormalities. However, the findings should be interpreted cautiously because of the case–control design, relatively small sample size, single-center setting, and the use of serological testing, which cannot distinguish active from past infection.

Although the combined inflammatory model demonstrated acceptable discriminatory performance, it is not intended to replace established diagnostic methods for active *H. pylori* infection, such as urea breath testing, stool antigen assays, or biopsy-based techniques. At present, the model may be more appropriately considered as a supportive research tool or preliminary screening approach rather than a standalone clinical diagnostic method.

Overall, the study highlights the importance of broader clinical evaluation of patients with *H. pylori* infection, including assessment of inflammatory status, metabolic regulation, and iron metabolism. Further large-scale longitudinal and interventional studies are warranted to clarify causal relationships and determine whether *H. pylori* eradication may improve these systemic outcomes.

## 6. Limitations

Several limitations of this research should be accounted for while assessing the results. To start, poorer internal validity may stem from the smaller sample size. Also, single-center sampling research may reduce external validity and introduce bias as well, especially in the context of case selection. Third, the assessed lifestyle included valued determinants but was vague and may have impacted the observed metabolic and hematological parameters, including, but not limited to, dietary intake, micronutrients, physical activity, socioeconomic status, and many other determinants. Exposure and outcome interrelationships may also be better appreciated with a cohort design. There are also other study-design-related limitations, such as case misclassification likely caused by reliance on serology in the diagnosis of *H. pylori* as this approach is not appropriate for active, confirmatory diagnosis, and discriminatory serology cannot be more specific about the case. Lastly, this research did not examine the effects of *H. pylori* eradication on the mentioned parameters. Future research directions include larger, distributed longitudinal studies with an interventional scope.

Another limitation is that the control group was defined based on apparently healthy status without confirmatory *H. pylori* testing for all participants, which may have introduced potential misclassification bias due to undetected asymptomatic infection.

## 7. Implications for Practice

The conclusions drawn by this investigation carry numerous practical implications for medical practice. First, the need for a broader evaluation of patients infected with *H. pylori* becomes obvious, encompassing not only symptoms related to the digestive tract but also assessing the condition of metabolism and hematology. In particular, the search for signs of disorders like insulin resistance or iron deficiency may prove crucial in risk groups.

Secondly, it can be assumed that *H. pylori* infection leads to the development of metabolic and hematological disorders such as insulin resistance or iron deficiency anemia. As a result, *H. pylori* treatment may have positive effects on metabolism and hematology. However, further research is needed to confirm this hypothesis. Thirdly, taking into account the public health perspective, the importance of diagnosing and treating H. pylori infection becomes apparent, especially in areas with a high prevalence of this disease.

## Figures and Tables

**Table 1:** Interpretation Categories of Area Under the ROC Curve (AUC) Values.

AUC (Area Under Curve)	Category
0.9–1	Very good
0.8–0.9	good
0.7–0.8	fair
0.6–0.7	fail
0.5 = 0.6	poor

**Table 2:** Demographic and Clinical Characteristics of the Studied Groups.

Variable	Diseased Group (n = 50)	Control Group (n = 50)	Test Statistic	*p*-Value
Age (years)	41.2 ± 13.1	36.3 ± 12.6	t = 1.89	0.062
BMI (kg/m^2^)	27.86 ± 5.61 (18.9–38.0)	26.0 ± 4.56	t = 1.81	0.074
Sex			χ^2^ = 1.44	0.230
Female	27 (54.0%)	21 (42.0%)		
Male	23 (46.0%)	29 (58.0%)		
Employment			χ^2^ = 1.97	0.160
Employee	41 (82.0%)	35 (70.0%)		
Student	9 (18.0%)	15 (30.0%)		
Marital Status			χ^2^ = 1.27	0.260
Married	39 (78.0%)	34 (68.0%)		
Single	11 (22.0%)	16 (32.0%)		
Smoking Status			Fisher’s Exact	<0.001 *
Smokers	17 (34.0%)	0 (0.0%)		
Non-smokers	33 (66.0%)	50 (100.0%)		
Fast Food Consumption (per week)				
None	8 (16.0%)	43 (86.0%)		
Once/week	17 (34.0%)	7 (14.0%)	Fisher’s Exact	<0.001 *
Twice/week	14 (28.0%)	0 (0.0%)		
Three times/week	11 (22.0%)	0 (0.0%)		

Note. Data are presented as mean ± standard deviation (range) or frequency (percentage). t = independent samples *t*-test; χ^2^ = chi-square test; * Statistically significant at *p* < 0.05; Fisher’s Exact = Fisher’s exact test (used when expected cell counts < 5).

**Table 3:** Hematological and Inflammatory Markers of the Studied Groups.

Variable	Diseased Group (n = 50)	Control Group (n = 50)	Test Statistic	*p*-Value
RBS (mg/dL)	112.36 ± 29.6 (80–220)	88.26 ± 7.64 (69–104.7)	t = 5.56	<0.001 *
HbA1c (%)	5.90 ± 1.14 (4.0–10.5)	5.4 ± 0.67 (3.6–6.4)	t = 2.66	0.009 *
CRP (mg/L) Median	5.45 (0.6–9.6)	2.75 (0.7–7.0)	U = 3.74	<0.001 *
ESR (mm/h) Median	22.5 (5–60)	11 (4–40)	U = 4.60	<0.001 *
Hemoglobin (g/dL)	12.3 ± 1.9 (7.5–16.1)	13.7 ± 0.93 (12.5–16.0)	t = 4.65	<0.001 *
Ferritin	11.9 ± 2.40 (6.0–15.8)	30.5 ± 3.00 (26–36)	t = 34.4	<0.001 *
WBC (×10^9^/L) Median	7.3 (2.1–23)	7.6 (4–12)	U = 0.63	0.528

Note. t = independent samples *t*-test; U = Mann–Whitney U test. RBS = random blood sugar; HbA1c = glycated hemoglobin; CRP = C-reactive protein; ESR = erythrocyte sedimentation rate; WBC = white blood cells. * Statistically significant at *p* < 0.05.

**Table 4:** Spearman Correlation Matrix Between Inflammatory, Glycemic, and Hematological Parameters (n = 100).

Variables		CRP (mg/L)	ESR 2ndhr (mm)
ESR 2ndhr (mm)	r	0.261 *	1
	*p*	0.009	-
RBS (mg/dL)	r	0.227 *	0.385 *
	*p*	0.023	0.000
HbA1c (%)	r	0.285 *	0.212 *
	*p*	0.004	0.035
Hemoglobin (g/dL)	r	−0.213 *	−0.254 *
	*p*	0.033	0.011
Ferritin	r	−0.180	−0.403 *
	*p*	0.073	0.000

Spearman rank correlation coefficient, * *p* < 0.05 significant, *p* ≥ 0.05 no significant.

**Table 5:** Logistic Regression Analysis of Significant Predictors of *H. pylori* Infection.

Variable	Univariate OR (95% CI)	*p*-Value	Multivariate OR (95% CI)	*p*-Value
CRP (mg/L)	1.460 (1.205–1.769)	<0.001 *	1.332 (1.041–1.705)	0.022 *
ESR (mm/h)	1.112 (1.056–1.171)	<0.001 *	1.086 (1.021–1.155)	0.009 *
Smoking status	2.984 (1.412–6.305)	0.004 *	2.216 (1.002–4.901)	0.049 *
Fast-food consumption	1.873 (1.294–2.712)	0.001 *	1.521 (1.012–2.287)	0.043 *

Note. OR = odds ratio; CI = confidence interval. Multivariate model adjusted for CRP, ESR, smoking status, and fast-food consumption. Hosmer–Lemeshow test: *p* = 0.512 (good model fit). Nagelkerke R^2^ = 0.47. No evidence of significant multicollinearity (all VIF values < 2.0). * Statistically significant at *p* < 0.05.

**Table 6:** Diagnostic Performance of CRP, ESR, and Their Combined Model for Predicting *H. pylori* Infection.

Parameter	Cutoff	Sensitivity (%)	Specificity (%)	Accuracy (%)	AUC (95% CI)	*p*-Value
CRP	≥6 mg/L	42.0	90.0	66	0.72 (0.61–0.82)	<0.001 *
ESR	≥12 mm/h	80.0	54.0	67	0.77 (0.67–0.86)	<0.001 *
CRP + ESR (combined model)	≥0.50 probability	60.0	76.0	68	0.82 (0.74–0.90)	<0.001 *

Note. AUC = area under the curve; CI = confidence interval. The combined model is based on logistic regression using CRP and ESR. A probability threshold ≥ 0.50 was used to classify positive cases. * Statistically significant at *p* < 0.05.

## Data Availability

The original contributions presented in this study are included in the article. Further inquiries can be directed to the corresponding author.
